# On the Size Effect of Gelation Kinetics in RF Aerogels

**DOI:** 10.3390/gels1020276

**Published:** 2015-12-10

**Authors:** Lorenz Ratke, Anna Hajduk

**Affiliations:** Institute of Materials Research, German Aerospace Center, DLR, 51170 Köln, Germany; E-Mail: ak.hajduk@googlemail.com

**Keywords:** gelation, aerogels, shear flow, aggregation kinetics, size effect

## Abstract

We experimentally and theoretically investigate the size effect of gelation using Resorcinol-Formaldehyde aerogels. We find a clear dependence of gelation time on the sample size under mild shear conditions. We developed a theoretical model based on Smoluchowski’s aggregation model adding, however, a growth term which accounts for the continuous growth of the colloidal particles while clustering happens. The model is solved analytically and agrees with our experimental observations for base catalyzed RF-gels.

## 1. Introduction

One essential step in the production of aerogels is the gelation of the colloidal solution starting from molecular precursors. Gelation is most often thought as being a consequence of aggregation and coalescence of nanometer to micrometer sized particles. Gelation is thought to have occurred once an aggregate of the colloidal particles builds a spanning cluster. Aging of the gels leads to further growth of particles, aggregation and stiffening of clusters by curvature dependent solution and re-precipitation. A kinetic description of gelation needs a source for particle motion and thus aggregation, like Brownian motion or some type of fluid flow. Most often, gel formation is described in the literature using a percolation approach or diffusion limited aggregation of single particles (DLA) or clusters (DLCA) [1,2,3]. Whereas percolation approaches essentially describe the gel structure, they cannot describe the kinetics, which is an advantage of DLA or DLCA methods. For instance, Halperin and co-workers [4] used an off-lattice DLA approach to describe the dry aerogel structure. They took a batch of particles taken from a Gaussian size distribution dropped into a given volume and allowed them to perform diffusive Brownian motion.

All models of percolation and DLA or DLCA, however, have the drawback that they fix the particle size from the very beginning of aggregation and gelation. The essential point of chemical kinetics in aerogel preparation, namely the starting from a solution of monomers, the building of dimers, trimers to oligomers and eventually to particles and their concurrent motion and aggregation to a fractal structure is rarely taken into account. There are a few models available like that of Yamamoto *et al.* [5], in which chemical reaction kinetics is used to describe the formation of *k*-mers, *k* ≥ 1, from monomers and combined with a population balance according to a classical Smoluchowski approach using a collision kernel of particles well-described in the literature for diffusive Brownian motion. They end up with a large set of non-linear equations, which are solved numerically. They are able to describe the particle growth rate, the size distribution and the specific surface area in agreement with experimental studies of the gel formation in polymeric Resorcin-Formaldehyde (RF)-gels with different catalyst concentrations. They do not investigate any size effect.

Independent of all approaches used, there always should be a size dependence, since the formation of a gel requires that a network of particles spans from one container wall to the other. The farther away the container walls, the longer it should take to establish a gel and, thus, the gelation time should increase. The scaling up of aerogels from granular materials of a few mL in volume to tiles of a few tens of centimeters or larger would profit from a knowledge of how gel formation depends on container size. It is astonishing that there is only one article found in the literature, which carefully deals with the size dependence using silica gels as a model substance.

Anglaret, Hasmy and Julien [6] studied experimentally and theoretically the gelation time as a function of container size for neutral and weakly base catalyzed Tetramethylorthosilicate (TMOS) gels. They find a size independent gel time for neutrally catalyzed gels and a gel time increasing with container size for base catalyzed gels. They compare their experimental observation with a fluctuating bond aggregation model (FBA), which is similar to a DLCA algorithm but needs a critical cluster concentration below which gelation is impossible. They also argue that a flexible bonding between particles in a cluster leads to a vanishing size dependence of gelation time from container size.

Any size effect in gelation of polymeric aerogels never was considered to the best of our knowledge. Therefore, we thought it might be interesting and worthwhile to test if base catalyzed resorcinol formaldehyde aerogels exhibit a size dependence experimentally. In order to describe the experimental investigations, we developed a continuum model based on the Smoluchowskis mean field approach [7] to describe the gelation kinetics. It turns out that our simple analytical model is able to describe the size dependence and we find, in agreement with the experiments, a weak, logarithmic dependence of gelation time on sample size.

## 2. Results

**Figure 1:**
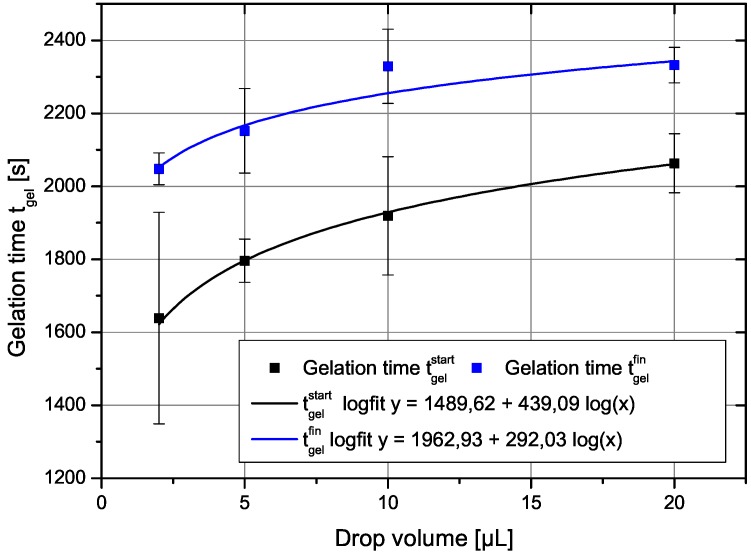
The two gel times defined above in the text as a function of drop volume. The drawn in curves are fit to a power law dependence on drop volume.

The gelation time depends clearly in a non-trivial way on the drop volume as shown in Figure 1. There, the two gel-times are plotted as a function of the drop volume. The gel time increases with drop volume in a manner suggesting a logarithmic dependence. The error bars show that the measurements of the gelation times have some inherent scatter. The drawn in curve is a power-law fit as shown in the legend. The two parameters of the fits would be for the start of gelation a=1528.8, b=0.0997, r=0.999 and for the end of the gelation process a=1966.3,b=0.062,r=0.953, with *r* as the correlation coefficient. A fit with a logarithmic dependence of gel time on drop volume yields almost the same result and is not shown here, since they would lie on each other with also almost identical correlation coefficients.

**Figure 2:**
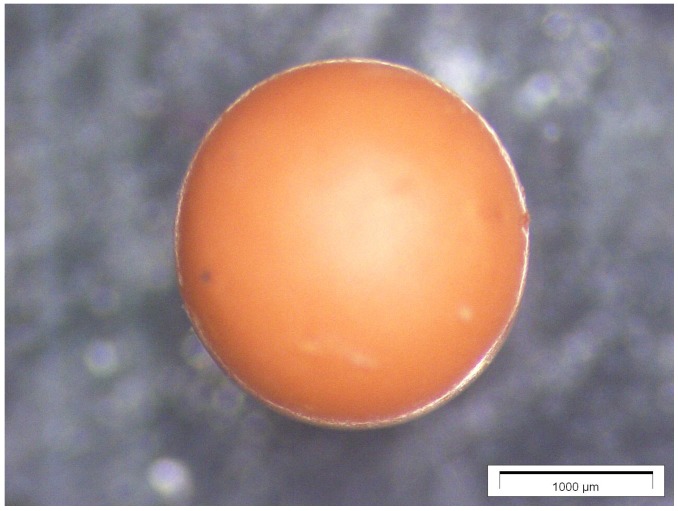
Microscopic image of a 10 μL RF-Aerogel droplet after drying under ambient conditions.

**Figure 3:**
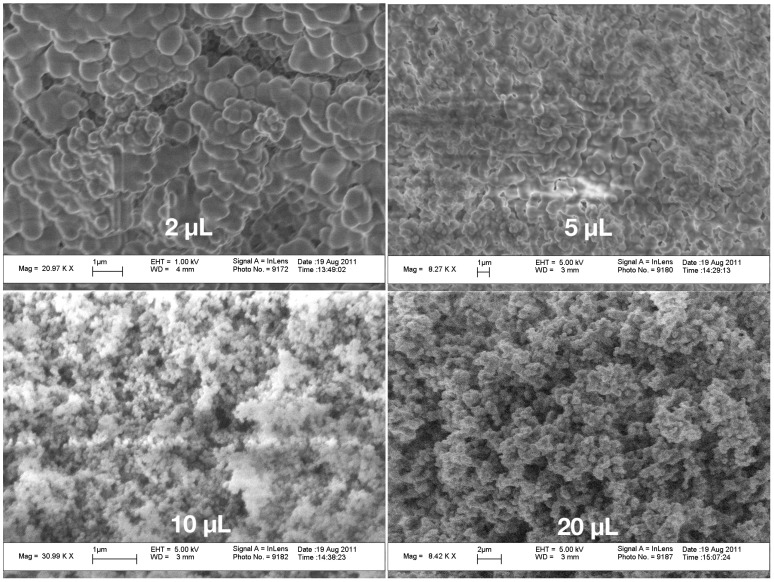
Scanning electron microscopic image of the fracture surface of a drop with 2 μL (**upper row left**), 5 μL (**upper row right**), 10 μL (**lower row left**) and 20 μL (**lower row right**).

During drying, the samples shrunk and attained their typical brown-ochre color as shown in Figure 2. They exhibited a different dry gel microstructure as shown in Figure 3 for all drop sizes used.

The SEM pictures show a largely aggregated and combined structure of the originally small particles, all connected by bridges. This appearance disappears as much as the drop volume increases. The drops with 10 and 20 μL volume already exhibit the typical aerogels structure of small particles aggregated in a network with pore space. There, the particles in the 10 μL samples are the smallest, below a micron in diameter. The particles in the network of the 20 μL samples are around a micron. Looking at the small drop volume suggests that the original primary particles which have aggregated were around a micron in size. One has to, however, take into account, that during drying, the samples shrunk and this affects the appearance of the network. The shrinkage of the 2 μL drops was about 58%, the 5 μL sample shrunk around 40%, those with 10 μL volume around 38% and the 20 μL drops only 28%. Thus, the deformation of the network during shrinkage was extremely different and may account for a few of the features seen in Figure 3. Nevertheless, the aggregation and compaction seems to decrease with increasing drop volume.

## 3. Theoretical Model

The data suggest that there is a non-linear dependence of gel time on droplet size, probably a logarithmic one. To the best of our knowledge such a relation is not reported in the literature. We therefore thought to develop an analytically tractable model reflecting specialties of our experimental set-up. We first have to take into account that the paraffin oil, which rotates in the bulb at low frequencies, fulfills a simple shear flow with an azimuthal velocity of $u_{\phi }=r\omega \sin\theta$ [8], with *r* as the radial coordinate in the bulb. The linear shear flow has a shear rate Γ=ωsinθ. Let us assume that the drops are at an angle of 45 ${}^{\circ }$C in the oil. We then have $\Gamma =\sqrt{2}\pi f$, with *f* the rotation frequency. In the suspended drop, a shear flow also exists, which is according to [9] described by a rotation period of ω=Γ/2. The shear rate inside the drop is then simplified as $\Gamma_{T}=\pi f/\sqrt{2}$.

### 3.1. Aggregation by Shear Flow

In order to describe gelation, we simply take the Smoluchowski equation [7], which describes aggregation like a kind of population balance: particles collide and aggregate forming bigger particles or clusters and particles vanish from a certain size class just due to this aggregation. If f(v,t)dv denotes the number density of particles with volumes in the size class v,v+dv the Smoluchowski equation reads

(1) $$\frac{\partial f}{\partial t}=\frac{1}{2}\int_{0}^{v}W\left(v,v-v^{\prime }\right)f\left(v^{\prime },t\right)f\left(v-v^{\prime },t\right)dv^{\prime }-f\left(v,t\right)\int_{0}^{\infty }f\left(v^{\prime },t\right)W\left(v,v^{\prime }\right)dv^{\prime }$$

with the collision kernel $W(v,v^{\prime })$, which is essentially the total scattering cross section of two particles having a volume *v* and $v^{\prime }$, multiplied with their relative velocity. The collision kernel $W(v,v^{\prime })$ defines the way drops coagulate by e.g., Brownian motion or shear flows [10,11,12]. We are not going to solve this equation for the given experimental conditions but will look for two issues which are easier to obtain then the full solution of the non-linear integro-differential Equation (1). First, the number density of colloidal particles inside the solution (transforming to a gel) n(t) can be obtained from integration of Equation (1) over the particle volume yielding the relation

(2) $$n\left(t\right)=\int_{0}^{\infty }\int_{0}^{\infty }W\left(v,v^{\prime }\right)f\left(v,t\right)f\left(v^{\prime },t\right)dv^{\prime }dv$$

If there is no growth of the particles due to chemical reactions, then the volume fraction of particles denoted as Φ is constant $\Phi_{0}$. The volume fraction and the average number density are always related as $\Phi =n\left(t\right)\bar{v(t)}$ via the average particle volume $\bar{v(t)}$. If there is no particle growth we have $\Phi_{0}=n\left(t\right)\bar{v(t)}$. Since a shear flow exists in the suspended drop, the colloidal particles forming in it will also feel the shear, as mentioned above, and we simply assume that they thereby make collisions due to the linearly varying velocity inside the bulb. The collision volume for shear flows can be simplified as [11]:

(3) $$W\left(v,v^{\prime }\right)≅\Gamma_{T}\left(v+v^{\prime }\right)$$

Inserting this into Equation (2) and integrating leads to simple expression for the change in number density

(4) $$\frac{dn}{dt}=-\Gamma_{T}n\left(t\right)\Phi_{0}$$

This equation is easily solved yielding the average cluster volume and the number density of clusters.

(5) $$n\left(t\right)=n_{0}\exp\left(-\Gamma_{T}\Phi_{0}t\right)$$

(6) $$\bar{v(t)}=\frac{\Phi_{0}}{n_{0}}\exp\left(\Gamma_{T}\Phi_{0}t\right)=v_{0}\exp\left(\Gamma_{T}\Phi_{0}t\right)$$

**Figure 4:**
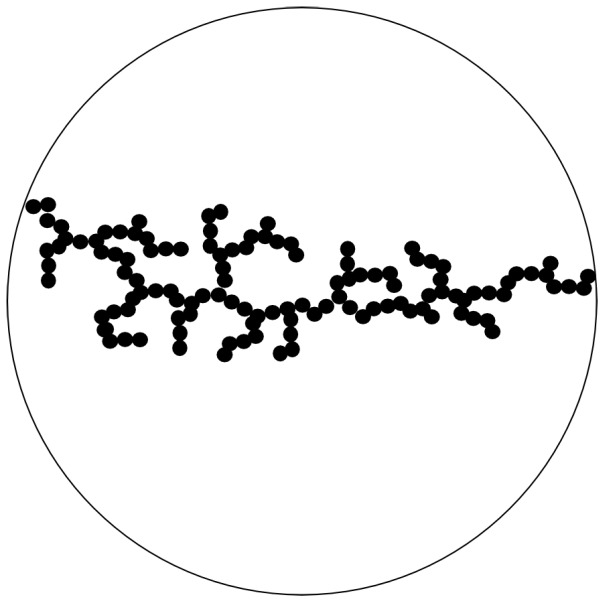
Gelation is assumed to occur, if a spanning cluster of particles having all the same size expands from one side of the suspended drop to the other.

It is important to notice that the average particle volume (aggregate volume) is unbounded. This is an essential feature of gelling systems [3,13]. Let us now define the gel time. This shall be done with the sketch shown in Figure 4.

All primary particles shall have initially a volume $v_{0}$. They aggregate to clusters. Gelation occurs when these small particles make a spanning cluster of $2R_{T}$. The number of particles in such a spanning cluster is

(7) $$N_{c}=\frac{2R_{T}}{v_{0}^{1/3}}=2{\frac{V_{T}}{v_{0}}}^{1/3}$$

Since this number is identical to $\bar{v(t)}/v_{0}$, we obtain

(8) $$N_{c}=\exp\left(\Gamma_{T}\Phi_{0}t_{gel}\right)$$

This immediately gives the gel time as

(9) $$t_{gel}=\frac{1}{\Gamma_{T}\Phi_{0}}\ln\frac{2V_{T}^{1/3}}{v_{0}^{1/3}}=a+b\ln V_{T}$$

This can be tested against the experimental values. Although a fit to the data (shown in Figure 1) looks very well, it is misleading, since the shear rate in Equation (9) also depends on drop volume. Inserting the above mentioned experimental power law relation, Equation (30), yields an expression like

(10) $$t_{gel}=\frac{V_{T}^{4/5}}{3.3\Phi_{0}}\ln\frac{2V_{T}^{1/3}}{v_{0}^{1/3}}$$

Such a relation cannot be fitted to the experimental results, since the gel time would depend almost linear on the drop volume, which is not the case. We nevertheless assume, that aggregation by shear collisions is an essential mechanism, since it gives a logarithmic dependence, but it is not sufficient to describe the experimental reality.

### 3.2. Concurrent Growth and Aggregation by Shear Flow

A better description of transformation from an RF solution to a gel should take into account, as mentioned in the introduction, that there is not only a size distribution of primary particles forming the network, but also a continuous growth of these, since there are abundant number of monomers, dimers *etc.* that can react with given oligomers and particles or clusters even after the first onset of gelation (aging period of gels). We take this into account by modifying the Smoluchowski equation with a growth term [12].

(11) $$\frac{\partial f}{\partial t}+\frac{\partial }{\partial v}\left(\frac{dv}{dt}f\right)=\frac{1}{2}\int_{0}^{v}W\left(v,v-v^{\prime }\right)f\left(v^{\prime },t\right)f\left(v-v^{\prime },t\right)dv^{\prime }-f\left(v,t\right)\int_{0}^{\infty }f\left(v^{\prime },t\right)W\left(v,v^{\prime }\right)dv^{\prime }$$

We again are not going to solve this equation for the size distribution, but first calculate the number density by integration of both sides of the equation with respect to the particle volume and then multiply both sides by the particle volume *v* and integrate again over the particle volume. This yields the number density and the volume fraction of particle clusters as it varies with time. Performing the integration leads to two ordinary differential equations.

(12) $$\frac{dn}{dt}=-\Gamma_{T}n\left(t\right)\Phi \left(t\right)$$

Instead of a constant volume fraction of RF-particles, we now have a time varying one

(13) $$\frac{d\Phi }{dt}=\int_{0}^{\infty }\frac{dv}{dt}f\left(v,t\right)dv$$

To evaluate these equations, we need an expression for the time varying change of particle volume dv/dt. For simplicity, we assume a constant volumetric growth of the particles with a chemical reaction of first order [14] through its interface area *A* at a rate $\nu_{0}$, meaning

(14) $$\frac{dv}{dt}=\nu_{0}A$$

Assuming that the particles remain spherical their surface area *A* can be expressed in terms of particle volume and Equation (13) be evaluated using $\Phi \left(t\right)=n\left(t\right)\bar{v(t)}$

(15) $$\frac{d\Phi }{dt}=\nu_{0}\alpha \Phi^{2/3}n^{1/3}$$

with α≈4.84. Solving Equations (12) and (15) together leads first to an expression for the number density as a function of volume fraction.

(16) $$n\left(\Phi \right)=n_{0}{\left(1-\frac{\Gamma_{T}}{4\alpha \nu_{0}n_{0}^{1/3}}\right)}^{3}$$

Setting $u=\Phi /\Phi_{s}$ as a new normalized variable of the volume fraction Φ with

(17) $$\Phi_{s}={\left(\frac{4\alpha \nu_{0}n_{0}^{1/3}}{\Gamma_{T}}\right)}^{3/4}$$

yields

(18) $$n\left(u\right)=n_{0}{\left(1-u^{4/3}\right)}^{3}$$

**Figure 5:**
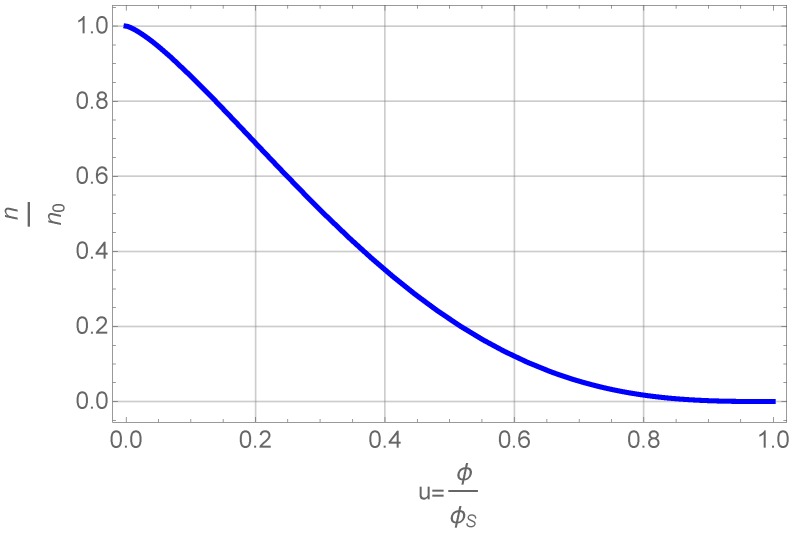
Number density of clusters as a function of normalized volume fraction.

The result is plotted in Figure 5.

This figure shows that the number density of primary particles, $n_{0}$, that can grow and aggregate decreases with volume fraction. The volume fraction itself is a function of time (see below) and thus we get the simple result, that there is a finite time after which all primary particles are part of clusters or even one cluster. Inserting this Equation (16) into Equation (15) and using again $\Phi =n\left(t\right)\bar{v}$ yields a differential equation for the volume fraction alone. Using again the normalized volume fraction *u* and refining the time sale using

(19) $$\theta =\frac{\nu_{0}\alpha n_{0}^{1/3}}{\Phi_{s}^{1/3}}t$$

the differential equation for the normalized volume fraction reads

(20) $$\frac{du}{d\theta }=u^{2/3}\left(1-u^{4/3}\right)$$

**Figure 6:**
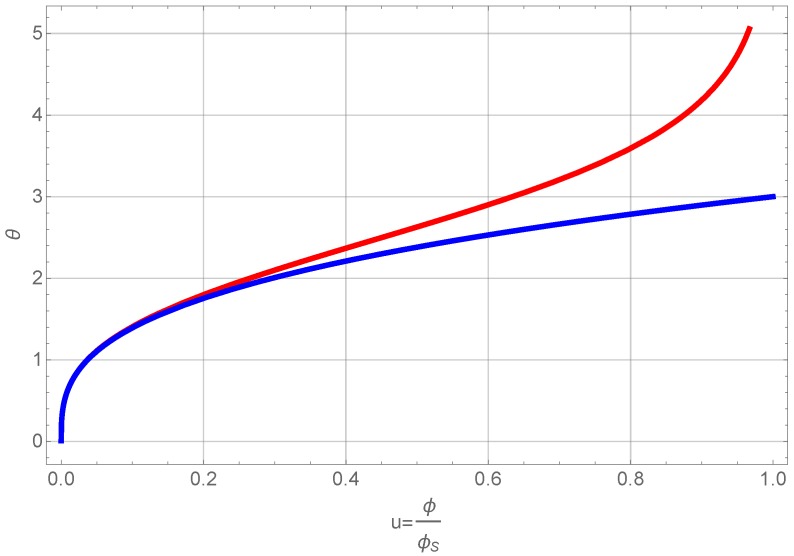
Normalized volume fraction of RF-particles as a function of scaled time *θ*. The blue curve shows the full solution and the red one the approximate one of Equation (21).

This equation can be integrated directly. The result is shown in Figure 6.

We omit presenting the full solution of Equation (20), but use only a series expansion at low volume fractions, since this allows us to further treat the problem analytically. A first order expansion of the normalized volume fraction as a function of normalized time gives

(21) $$u≅\frac{\theta^{3}}{27}$$

A comparison of the full solution and the approximate one is also presented in Figure 6 showing that the approximation is valid for θ<2. Integrating Equation (12) directly and using the normalized time leads to an expression of the number density

(22) $$n\left(\theta \right)=n_{0}\exp\left(-4\int_{0}^{\theta }u\left(\theta \right)d\theta \right)$$

and similarly one can derive for the average particle volume the relation

(23) $$\bar{v(\theta )}=\frac{\Phi_{s}}{n_{0}}u\left(\theta \right)\exp\left(4\int_{0}^{\theta }u\left(\theta \right)d\theta \right)$$

We now have to define an expression of the gelation time for aggregates consisting of particles with different sizes.

**Figure 7:**
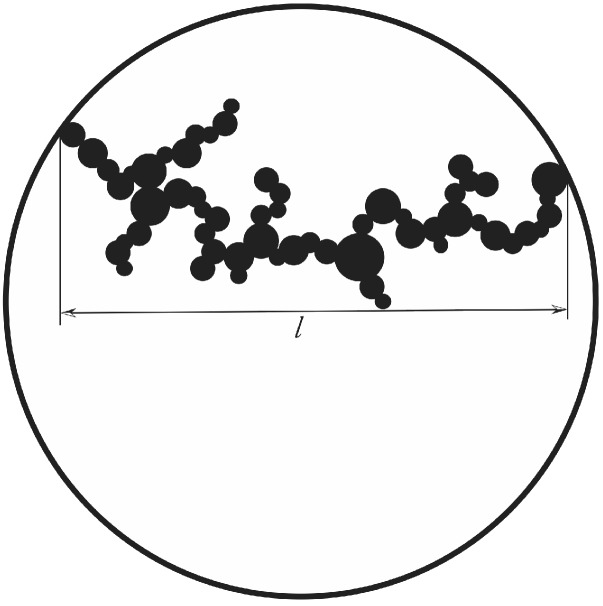
If the particles for during aggregation the cluster consist of particles with different size. Still gelation is assumed to occur, if a spanning cluster of particles now having all the different sizes expands from one side of the suspended drop to the other.

A sketch of such an aggregate is shown in Figure 7. We can define that for a cluster of length $\ell \approx V_{T}^{1/3}$ the number of particles at the gelation time $t_{g}$ is given by

(24) $$N_{c}={\left(\frac{V_{T}}{\bar{v(t_{g})}}\right)}^{1/3}$$

The number can be calculated from the number density n(t) and the suspended drop volume $V_{T}$ to be $N_{c}=n\left(t_{g}\right)V_{T}$. Putting these equations together leads to an expression for the gel time

(25) $$V_{T}^{-2}=n{\left(t_{g}\right)}^{3}\bar{v(t_{g})}$$

Inserting the above expressions for the number density and the average particle volume into this expression using u(θ), the approximate result given in Equation (21) gives the relation (using normalized variables)

(26) $$V_{T}^{2}n_{0}^{2}\Phi_{s}=\frac{27}{\theta_{g}^{3}}\exp\left(\frac{2}{27}\theta_{g}^{4}\right)$$

with $\theta_{g}$ as the obvious definition of a scaled gelation time. Although this equation can be formally solved for $\theta_{g}$ using the Lambert function, we do make a simplification which allows a direct comparison with the experimental data. Taking on both sides of Equation (26), the logarithm allows one to give an expression for the gel time as

(27) $$\theta_{g}≅{\left(2+\ln(V_{T}^{2}n_{0}^{2}\Phi_{s}\right)}^{1/4}$$

In this expression, we approximated the term stemming from the logarithmic form of Equation (26), namely $\ln(27/\theta_{g}^{3})≅-2$ being valid for normalized gel times between four and six. The result in Equation (27) can be compared with the experimental results after transformation to non-normalized coordinates and using the power law relation between the shear rate and the drop volume. This gives the expression

(28) $$t_{g}=\frac{{\left(an_{0}^{1/3}\alpha \nu_{0}\right)}^{1/4}V_{T}^{1/5}}{\alpha \nu_{0}n_{0}^{1/3}}{\left[2+\ln\left(bn_{0}^{2}V_{T}^{2}{\left(n_{0}^{1/3}V_{T}^{4/5}\alpha \nu_{0}\right)}^{3/4}\right)\right]}^{1/4}$$

with a,b numerical constants from the mentioned power law relation. To make a fit to the experimental data, we have to take into account, that the model assumes that a primary particle density $n_{0}$ exists from the beginning. This, however, is in reality not the case. At the beginning of the experiment, the solution contains resorcinol, formaldehyde, water and the catalyst. The monomers have to react to build first dimers, trimers and oligomers. Before they can be treated as distinct primary particles, which are entrained in the shear flow field, it takes a certain incubation time $t_{incub}$, as is well known for silica gels and discussed by Iler [15] or in nucleation theory as discussed by Kelton and Greer [16]. We therefore add to the above derived expression an incubation time and simplify it further by neglecting the expression in square brackets (an evaluation shows, that the logarithm is a very slowly varying function with drop volume and almost constant to around 2.8 for the drop volumes used and a primary particle density in the range of 10${}^{12}$ to 10${}^{16}$ and growth rates $\nu_{0}$ in the range of 0.1 to 1 nm/s.). This leads us to our final equation

(29) $$t_{g}=t_{incub}+\frac{{\left(1.5an_{0}^{1/3}\alpha \nu_{0}\right)}^{1/4}V_{T}^{1/5}}{\alpha \nu_{0}n_{0}^{1/3}}=t_{incub}+cV_{T}^{1/5}$$

**Figure 8:**
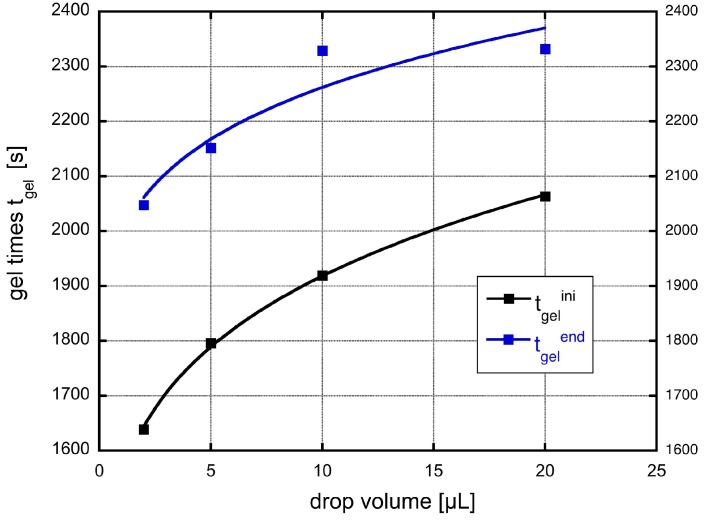
Measured gel-time compared with the theoretical expression given in Equation (28).

A fit of the experimental data to the theoretical expression is shown in Figure 8. Here, we have used the original drop volumes given in μL.

We yield for the incubation times related to the onset of gelation and the final gelation point two different values, $t_{incub}=920$ and $t_{incub}=1530$ s, respectively. The constant *c* can be re-calculated to yield a value for the product $\nu_{0}n_{0}^{1/3}$ a value of 4$10^{-5}$. This is for instance fulfilled for a reasonable initial concentration of primary particles of $n_{0}=10^{-15}$ m${}^{-3}$ and a growth rate $\nu_{0}$ of 0.1 nm/s. Such a growth rate would mean that the particles grow in 1000 s to a size of 100 nm, which sounds reasonable and comparable to the SEM figures shown. The result also shows, that both effects are important, the growth, which leads in Equation (28) to the dependence on shear rate to a power of 1/4 and the shear coagulation itself, reflected in the shear rate dependence. The microstructures shown in the SEM pictures of Figure 3 also reflect the issue that, in the small droplets, the shear rate was higher than in the larger ones and, thus, they are more compact compared to those in which aggregation took place under mild shear conditions. To calculate the incubation time, another model for the chemical reactions kinetics would be needed.

## 4. Conclusions

The size effect of gelation in polymeric aerogels was investigated experimentally with a new technique leading to shear flow assisted aggregation of primary particles. The theoretical modeling revealed that shear induced aggregation based on the mean-field model of Smoluchowski is able to describe essential features of the experimental observations. The result shows that the size dependence is weakly depending on the shear rate or the sample volume. Nevertheless, the result might be important for experimental situations, in which stirring of a solution is usually preceding gelation and especially to situations in which the stirring persists till the onset of primary particle formation and then already induces aggregation and cluster formation.

## 5. Materials and Methods

**Figure 9:**
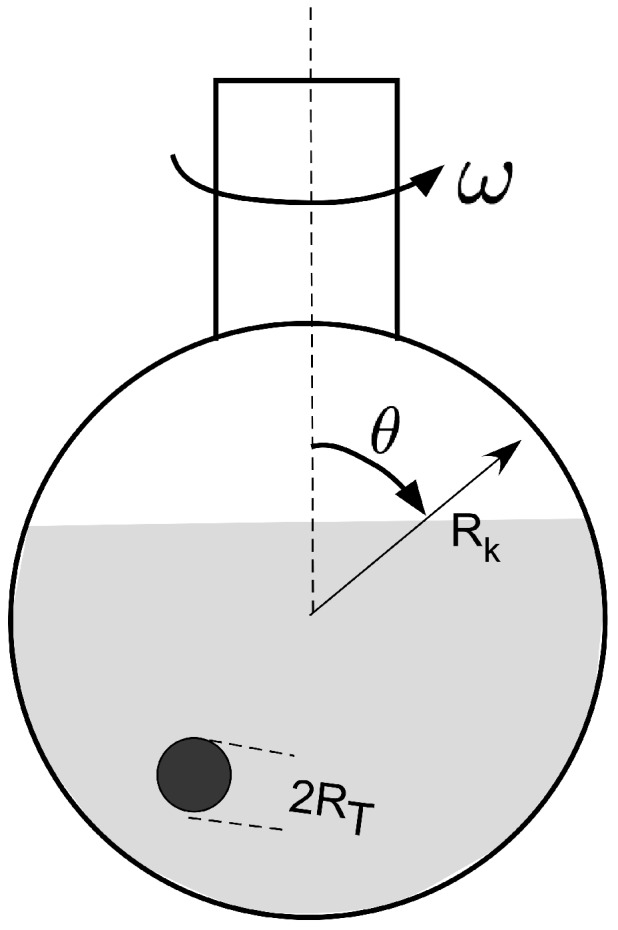
Drops of different volume (leading to different drop radii $R_{T}$), were placed in a rotating bulb of radius $R_{K}$ which was inclined at an angle *θ* to the vertical and rotated with different angular frequencies ω=2πf.

For this study, we used resorcinol-formaldehyde gels using 1.57 g resorcinol (R) (99% purity, Merck) mixed with 5 g deionized water (W) and 24.3 g formaldehyde (F) (24%, Merck) and 0.011 g Na${}_{2}$CO${}_{3}$ as a catalyst (C). The R/C ratio is 1515. In contrast to Anglaret *et al.* [6], we did not use cylinders of different aspect ratio to study the size effect but decided to use a spherical shape. This at least has a clear definition of size. We used a special experimental arrangement shown schematically in Figure 9.

In a bulb shaped glass container with two cylindrical inlets, paraffin oil was filled. The oil was heated to 80 ${}^{\circ }$C in a water bath controlled by a thermostat to about 0.1 ${}^{\circ }$C accuracy. Then, the freshly prepared resorcinol-formaldehyde solution was inserted as a drop using Eppendorf pipettes with stainless steel needles allowing to exactly control the drop volume. Since a small difference in density between the solution and the drop could not be avoided, the bulb was rotated to impede settling. If drops still touched the wall, these were not taken into account for the measurement of gel time. The rotation frequency had to be varied with the drop size as shown in Table 1. The variation of rotation speed was necessary since, at larger rotation speeds, larger droplets deform in the oil bath to a non-spherical shape. The viscous drag on the larger drops is larger than that on smaller ones so that the speed of rotation had to be lowered without deformation of the drops. Thus, we have the experimentally difficult situation that the rotation speed varies with the droplet volume in a non-linear fashion. A fit of the rotation frequency with the drop volume reveals a power law relation of the type

(30) $$\Gamma_{t}=\xi V^{-4/5}$$

with *ξ* a numerical constant depending in the measure used for the volume. If the volume is measured in μL, we obtain ξ=3.3. The definition of the shear rate $\Gamma_{t}$ is given below. For the theoretical analysis to be performed below, we have to take this dependence into account.

**Table 1:** Rotation frequencies of the bulb as varying with the volume of the suspended drop.

Drop Volume in μL	Rotation Frequency in rpm
2	50
5	24
10	16
20	8

**Figure 10:**
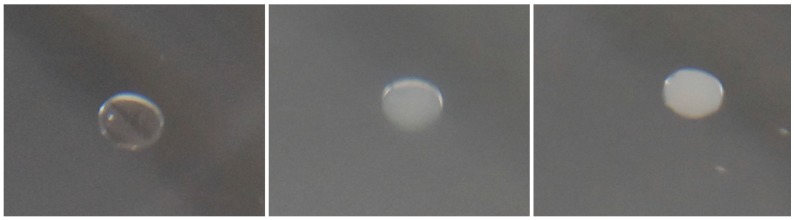
The left figure shows the drop freshly immersed in oil, in the middle the onset of gelation can be seen, denoted as $t_{g}^{start}$, and the right figure shows the final gelation step. The drop has attained a stable size. This time is denoted as $t_{g}^{fin}$.

The gelation time was measured using a stopwatch and carefully looking at the immersed drop. On gelation, the initially clear solution becomes first turbid and then opaque, grey-white. This is shown in Figure 10. We therefore denoted two different gel-times. The change from clear to turbid we call the starting of gelation. This time was recorded as $t_{g}^{start}$. The change to opaque was recorded as the finishing time of gelation $t_{g}^{fin}$. This is a simple way to characterize the onset of gelation. Classically, especially in polymer physics, a rheometer is used to measure for instance the viscosity or the storage or loss modulus [17,18,19,20] as a function of time at constant shear rate or stress and a substantial change of their values can be identified as gelation to happen. Recently, a non-invasive optical method was applied for silica gels [21]. These methods also do not give an exact time for gelation, but the change in viscosity or storage modulus occurs always over a finite time interval and therefore also would lead to an onset time of gelation and a finish time. We therefore think that our method is very simple but sufficiently accurate, especially since other methods are hardly applicable in our experimental set-up.

We repeated all experiments at least ten times with ten drops of the same volume preparing for each drop a new solution. We were able to study the gelation of four different drop volumes, namely 2, 5, 10 and 20 μL. Larger drops could not be studied with this technique, since they deform too much in the flow field of the oil bath.

After gelation, the samples were aged at 80 ${}^{\circ }$C in the oil bath for one hour and then washed with heptane to get rid off the paraffin oil. Finally, the samples were subcritically dried in a furnace at 80 ${}^{\circ }$C. The dry samples were looked at in a stereo microscope (Leitz, Wetzlar) and were broken with a scalpel. Scanning electron microscopy (Zeiss Merlin, Germany) was performed on fractured surface of the dry drops after sputtering with a thin gold layer to avoid charging artefacts.
